# Peripheral vascular access as exclusive access mode in pediatric intensive care unit

**DOI:** 10.3389/fped.2023.1259395

**Published:** 2023-10-09

**Authors:** Sonya Hayes Armstrong, Shantaveer Gangu, Alina Nico West, Thomas Spentzas

**Affiliations:** ^1^ Pediatric Intensive Care Unit, Le Bonheur Children’s Hospital, Memphis, TN, United States; ^2^ Division of Pediatric Critical Care Medicine, University of Tennessee Health Science Center, Memphis, TN, United States

**Keywords:** central venous catheter, central line-associated bloodstream infection, central venous access device, extended dwell catheters, Michigan appropriateness guide for intravenous catheters, midline catheters, peripheral intravenous catheter, peripherally inserted central catheter

## Abstract

**Introduction:**

The type of vascular access (central or peripheral) in pediatric critical care depends on several factors, including the duration of treatment, the properties of the medication (osmolarity or vesicant), and the need for central pressure monitoring. The utilization of peripheral intravascular catheters (PIVCs) has shown a notable increase in the number of patients being treated. Extended dwell or midline catheters are another peripheral access option in addition to PIVCs. However, there are currently no established guidelines on their placement.

**Objectives:**

The aim of this study is to estimate the duration of dwell time for PIVCs, analyze the specific parameters affecting it, and develop recommendations for switching to extended dwell and midline catheter placement as an alternative to peripheral access.

**Methods:**

The study enrolled patients aged 0–18 years admitted to the pediatric intensive care unit (PICU) for over 24 h and managed with peripheral access only over 2 years (2019–2021).

**Measurements and main results:**

A total of 484 patients met the specified criteria. Patients who had peripheral access exhibited a lower PRISM score and a shorter length of stay in the PICU, with mean values of 18 (SD: 8.5) and 9.5 (SD: 6.4) days, respectively, compared with patients who had central access with mean values of 8.9 (SD: 5.9) and 5.7 (SD: 3.6) days, respectively. The PIVC dwell time was found to be 50.1 h (SD: 65.3) and required an average of 1.6 insertion attempts. Patients with three or more insertions exhibited an increased odds ratio of 5.2 (95% CI: 3.1–8.5) for receiving an extended dwell or midline insertion. Increased dwell time was associated with female gender, 59.5 h (*P *< 0.001), first attempt insertion, 53.5 h (*P *< 0.001), use of 24 Ga bore, 56.3 h (*P *= 0.04), left-sided insertions, 54.9 (*P *= 0.07), less agitation, 54.8 h (*P *= 0.02), and less edema, 61.6 (*P *< 0.001). Decreased dwell time was associated with the use of vancomycin infusion at 24.2 h (*P *< 0.001) and blood transfusions at 29.3 h (*P *< 0.001).

**Conclusions:**

Extended catheters last longer than PIVCs in PICU patients. Extended catheter placement requires consideration of the length of treatment, as well as the overall body edema, the level of the patient's restlessness, and the need for vancomycin infusion or blood transfusions, as these factors reduce PIVC dwell time and expose the patients to painful insertions. For such cases, an extended dwell catheter may be a better option, even if the projected treatment time is less than 6 days.

## Introduction

Majority of the patients in pediatric critical care require a form of vascular access (VA). In contemporary healthcare settings, the insertion of peripheral intravascular catheters (PIVCs) by bedside nurses remains the most commonly performed procedures. Many modern pediatric intensive care unit (PICUs) use ultrasound and near-infrared technologies combined with anxiolysis or anesthesia, interventional radiology, and specialized VA teams to facilitate vascular access (1, 2). However, access remains an invasive, painful, and complex procedure.

**Figure 1:**
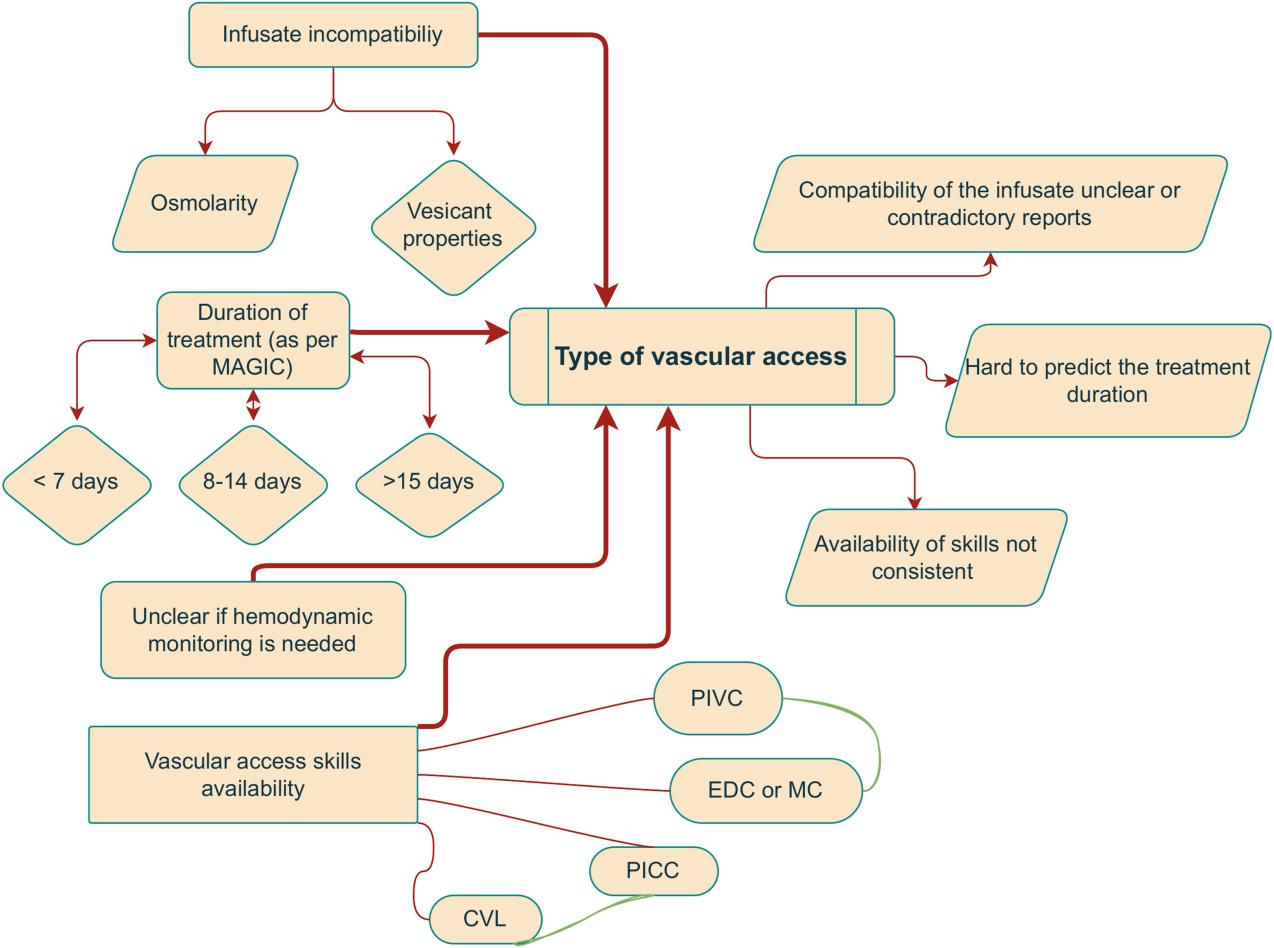
The decision for vascular access in the PICU is multifactorial and admittedly confusing. The flowchart shows that it gets more complicated: on the left are the most frequent “indications,” and on the right is the associated ambiguity.

The Michigan Appropriateness Guide for Intravenous Catheters (MAGIC) is an expert consortium recommending the type of intravenous catheter (central or peripheral) for adult patients in critical care settings (3). Following the recommendations of MAGIC, the miniMAGIC consortium adjusted the guidelines for children (4). In their review, the duration of treatment and the need for hemodynamic monitoring are the most critical parameters. There are more factors to be considered since the dwell time of PIVCs depends additionally on other factors such as agitation, edema, or characteristics of the infusion, including vancomycin treatment or blood transfusion, as described in previous studies (5–7) (Figure 1).

Unstable critical care patients most often need a central catheter placement, and such central venous catheters (CVCs) are placed by the ICU team (8). In relatively less urgent situations, a peripherally inserted central catheter (PICC) line might be an option if there is sufficient time available. PICC can be used for administering intravenous medications with high osmolarity or vesicant infusions. However, PICC may not accurately transmit the central pressure (9). Midline catheters (MC) are often characterized by their shorter length compared with PICC, as well as their peripheral placement resulting in the tip not being positioned centrally (10). Extended dwell catheters (EDC) is of shorter length than MC, but longer than PIVC (11).

This study aims to describe the baseline dwell time of PIVCs and the factors that increase or decrease it. Knowing the average PIVC dwell time in an edematous or agitated patient or those requiring different treatment necessities can assist in determining whether placing a longer-lasting EDC or MC earlier is beneficial, thus providing more specific recommendations.

## Materials and methods

The study included all patients admitted to the pediatric intensive care unit (PICU) between 1 January 2019 and 31 December 2021, who stayed at least 24 h and were managed without central access. Electronic medical records were retrospectively utilized to collect the data. The study titled “Longevity of Peripheral Intravascular Catheter” was approved by the University of Tennessee on 14 July 2022, 22-08810-XP. The need for informed consent was waived. The University adheres to the ethical standards governing human experimentation and the Helsinki Declaration of 1975.

The analysis encompassed patient information, including age, gender, race, weight, location of the access, and number of PIVCs, as well as factors that affected dwell time, such as generalized edema. Edema was assessed by employing skin indentations or with a fluid overload equation. The fluid overload equation is *(current weight − admission weight)/admission weight* (12, 13). Using PIVC for hydration, sedation, antibiotics, inotropes, and nutrition was recorded. The agitation (or hyperkinetic delirium) was also extracted.

Dwell time was computed from the documented insertion time until the recorded removal time of the PIVC. The reasoning for removal was noted as “end of treatment” or “failure” due to clotting or dislodgment (infiltration). In case of an infiltration or clotting, it needs to be reported to the institution's quality improvement monitoring. End-of-treatment removal might refer to a particular treatment conclusion and not necessarily that all intravascular treatments have finished: in case that PIVC was inserted to facilitate a transfusion and subsequently became clotted at the end, it can be removed and documented as the “end of treatment.” Thus, to avoid confusion with the uncertainty created by the definition of the “end of treatment,” dwell time was defined as the time from insertion to removal for any reason.

The functional dwell time was defined from insertion to removal because of a documented non-functioning, such as clotting or dislodgment (infiltration). It was computed with non-parametric survival Kaplan–Meier methods: it did not include PIVCs removed with documentation “end of treatment.”

The analysis was performed using IBM SPSS Statistics for Windows, version 29.0. Armonk, NY, USA. The data are presented as means (SD), and the associations are assessed using ANOVA for parametric and chi-square tests for non-parametric variables. The comparisons were performed under *a priori* assumption, and the significance *α* was set at .05. The BF_10_ and Kaplan–Meier were validated using JASP version 0.17.1 (2022) and Jamovi version 2.3 (2022).

## Results

**Figure 2:**
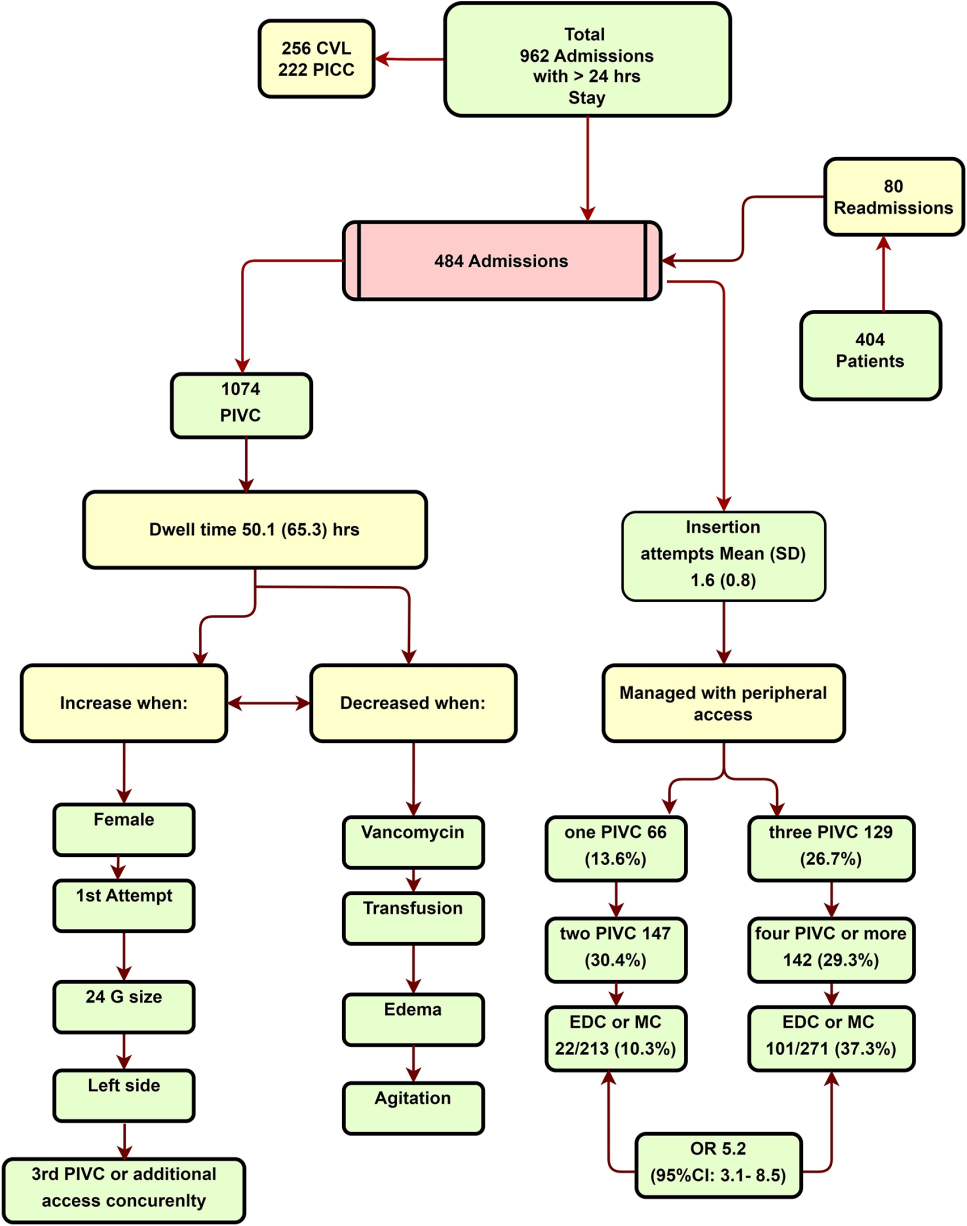
CONSORT diagram.

Figure 2 and Table 1 describe the characteristics of the enrolled patients. The study reviewed all the admissions in the PICU that lasted for 24 h or more. A total of 478 patients were excluded from the analysis, with 256 patients having central venous catheter (CVC), and 222 patients having PICC. The patients in the CVC and PICC group (central access group) had a higher level of sickness compared with those managed with peripheral access. The mean PRISM and PICU LOS for the central access group were 18 days (SD: 8.5) and 9.5 (SD: 6.4) days, respectively. In comparison, the peripheral-only access group had 8.9 (SD: 5.9) and 5.9 (SD: 3.6) days, *P *= 0.01 (BF_10 _= 4.25 × 10^+66^ = 0.001 (BF_10 _= 2.12 × 10^+22^). The central access placement in cases where there was only an indication of difficult vascular access was reported in 14.1% 36/256 (14.1%). The peripheral-only group included 484 admission records (404 patients) with 1,074 PIVC insertions (see Figure 2). In total, 80 patients were readmitted, with 22 readmissions occurring from home and 58 from the institution.

**Table 1:** Patients' demographics and clinical characteristics.

Age (years)	*N* = 404	Percent (%)	Time (h)	SD	*P-*value
0–1	105	26	58.6	70.3	0.17
2–5	85	21	56.3	72.6	* *
6–12	83	20.5	46.3	73.4	* *
13–18	131	32.4	45.3	58.9	* *
Weight	*N* = 404				0.48
	35.5 kg	SD: 29.5			* *
Race	*N* = 402	Percent (%)	Time (h)	SD	0.31
Black	185	46	45.7	61.3	* *
White	162	40.3	51.7	65.6	
Hispanic	15	3.7	60.8	76.5	
Other	40	10	46.7	53.8	
Sex	*N* = 402	Percent (%)			<0.001*
Males	239	59.5	42	55.6	
Females	163	40.5	59.5	73.5	
PIVCs per admission	*N* = 484	Percent (%)	Time (h)	SD	<0.001*
1st PIVC	66	13.6	40.8	60.7	
2nd PIVC	147	30.4	42.9	72.1	
3rd PIVC	129	26.7	57.8	88.3	
4th PIVC or more	142	29.3	81.0	81.3	
Agitation +	77	15.9	46.6	76.2	0.02*
Agitation −	407	84.1	54.8	62.8	
Edema +	426	88	52.4	62.4	<0.001*
Edema −	58	12	61.6	82.5	
Attempts	Mean: 1.6	SD: 0.8			
	*N* = 1,074	Percent (%)	Time (h)	SD	<0.001*
1st Attempt	668	66.2	53.5	69.5	
2nd Attempt	195	18.2	48.4	65.6	
3rd Attempt +	211	19.6	40.7	48.6	
Size of PIVC	*N* = 1,020	Percent (%)	Time (h)	SD	0.04*
16G	5	0.50%	30.8	42	
18G	79	7.70%	52.4	77	
20G	220	21.60%	39.7	52.8	
22G	403	39.50%	54.3	65.7	
24G	275	27%	56.3	72.1	
IO	11	1.10%	15	4.2	
Other	27	2.60%	54.8	85.9	
Location	*N* = 1,021	Percent (%)	Time (h)	SD	<0.001*
Antecubital	491	48	50.2	63.5	
Hand	332	32.5	47.7	63.2	
Foot	171	16.8	61.9	80.5	
Other	27	2.6	36.8	49.1	
Laterality	*N* = 1,019	Percent (%)	Time (h)	SD	0.07
Left	492	48.3	54.9	73.4	
Right	527	51.7	47.3	59	

* Indicates significance at the level *α* < 0.05.

The mean age of the patients included in the study was 4.52 years (SD: 4.16). In particular, the study included a total of 105 (26%) patients, 0–1 year of age (YOA), 85 (21%) patients, 2–5 YOA, 83 (20.5%) patients, 6–12 YOA, and 131 (32.4%) patients, 13–18 YOA. The dwell times were not statistically different between the age subgroups (*P *= 0.17), Table 1. The age distribution of the study sample was similar to the age distribution of patients admitted to the PICU at the institution.

The mean weight was 35.5 kg (SD: 29.5). A multiple linear regression was used to test if Age and Weight significantly predicted the Dwell time. The overall regression was not statistically significant [*R*^2^ = 0.01, *F*(df_1 _= 2, df_2 _= 481) = 2.36, *P* = 0.09].

The PIVCs lasted an average of 50.1 h (SD: 65.3), while female patients showed a slightly longer dwell time over males 59.5 vs. 42 h, *P = *0*.*01 (Table 1).

There was no significant difference found between races, *P *= 0.31. In total, 66 (13.7%) admissions were managed with one PIVC insertion. However, 129 (26.7%) admissions needed three PIVCs, and 142 (29.3%) of the admissions required four or more PIVCs. There were 1.6 insertion attempts per patient (SD: 0.80). PIVCs placed on the first attempt lasted longer than those required multiple attempts, with a mean of 53.5 h (SD: 69.5) (*P *= 0.01), as well as the 24 Ga bore than larger bore catheters, lasting 56.3 h (*P *= 0.01). The incidence of reported infiltration was rare, accounting for just six out of the 1,074 (.5%) PIVC insertions. The potential under-reporting of the incidence could be attributed to the laborious process of the reporting system.

Among the admissions that required three or more PIVC insertions, 101 out of 271 (37.3%) required EDC or MC in comparison with patients who had one or two PIVC insertions [(22/213 (10.3%), *P < *0.001, odds ratio (OR) 5.2 (95% CI: 3.1–8.5), BF_10_ = 4.92 × 10^+8^].

PIVCs that were inserted later in the admission course had a longer duration (Table 1) and were highly correlated with EDC or MC insertion at Pearson *ρ *=* *0.89.

PIVC inserted on the left side exhibited longer dwell times. In addition, there was a trend for the foot PIVCs to demonstrate a statistical significance, with a mean dwell time of 61.9 h (SD: 80.5), *P *= 0.08 (trend). Edema was reported in 58 out of 484 (12%) admissions, and the mean dwell times were found to be worst for this group at 46.6 h (SD: 76.2) compared with those without edema at 54.8 h (SD: 62.8), *P *= 0.01.

PIVCs were utilized predominantly for fluid administration (432, 89.3%), sedation (219, 45.3%), and administration of antibiotics (193, 39.9%). The use of sedation medications was associated with a prolonged mean dwell time of 58.5 h (SD: 47.70), *P *= 0.01. The group did not include patients who were administered total parenteral nutrition (TPN) with dextrose concentration higher than 12.5% or hypertonic saline solutions with higher than 2% concentration via the PIVC, while inotropes were infused in a few cases. The total number is small, 32/484 (6.6%), and the duration of these cases is less than 24 h. The study found no difference in the dwell time of the PIVCs in any of the above categories.

Vancomycin infusion and blood transfusion significantly decreased the mean dwell time to 24.2 h (SD: 34.7) and 29.3 h (SD: 41.7), respectively, at *P *< 0.001. Inconsolable agitation or delirious behavior was documented in 77 patients. The mean dwell times were shorter at 46.6 h (SD: 76.2) than at 54.8 h (SD: 62.8).

**Figure 3:**
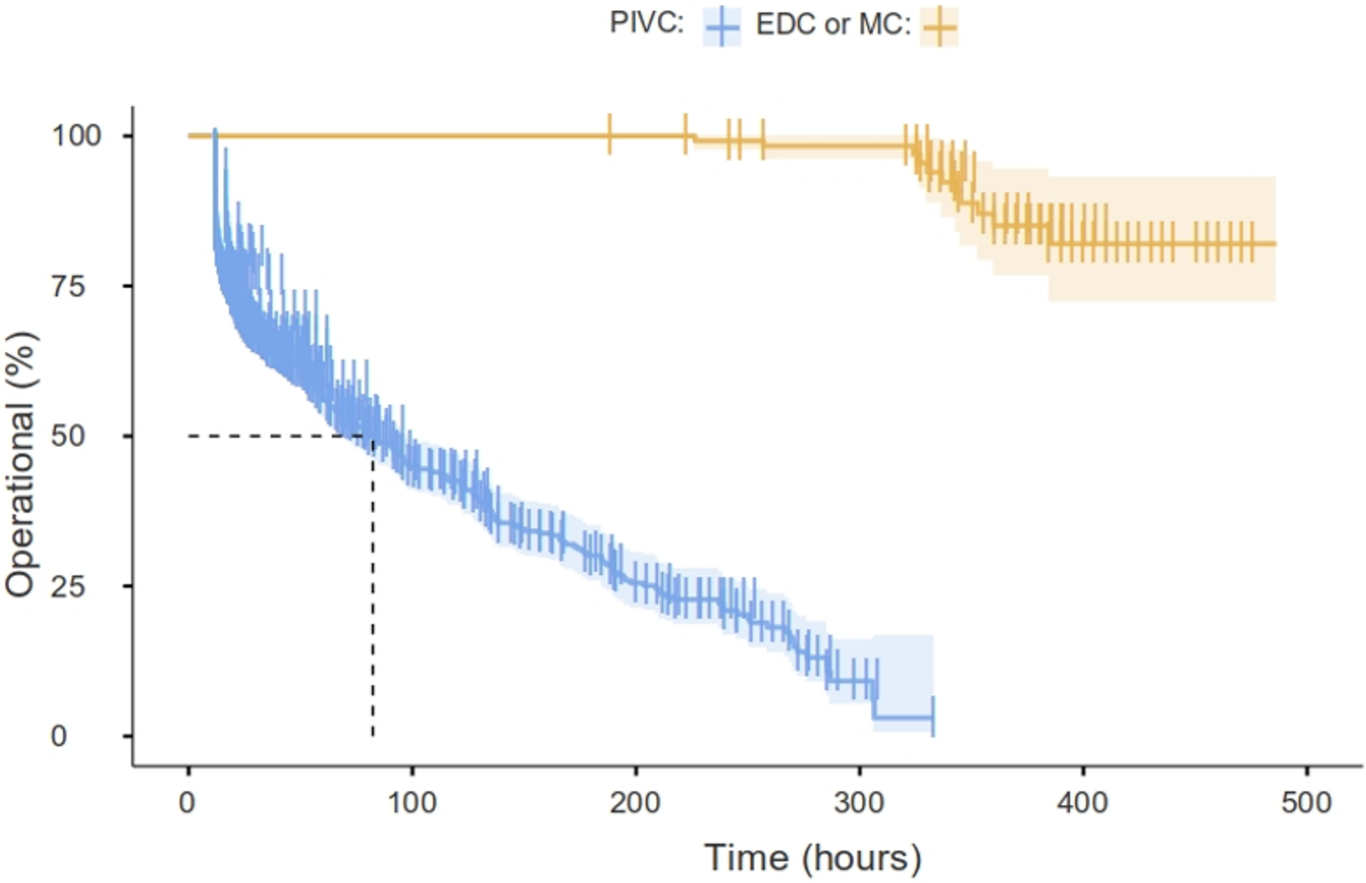
Percent operational PIVC vs. EDC or MD overtime (Kaplan–Meier). The median of the PIVC survival to failure time is 82 h (67.9–97.2), and it is not possible to estimate the EDC or MD dwell time as they remain in place longer than the patient’s PICU stay (the patients were discharged to the pediatric floor with a functional catheter). This time is called “functional dwell time” (insertion to not functioning) to distinguish it from the dwell time computed from insertion to removal for any reason (not functioning or reported as end of treatment).

Kaplan–Meier curves are used to compare the PIVC with EDC or MD, as shown in Figure 3. The long rank value is *X*^2 ^= 205 (*P *= 0.01). There is a 460/1,074 (42.8%) PIVC failure rate (reported as occluded or infiltrated) vs. 11/123 (8.9%) for EDC or MC (BF_10 _= 2.96 × 10^−13^). The median survival time for the PIVC until failure (functional dwell time) is 82 h (67.9–97.2). However, it is not possible to estimate the EDC or MD dwell time as they remain in place longer than the patient’s PICU stay (the patients were discharged to the pediatric floor with a functional catheter).

## Discussion

Despite the significant number of critically sick children managed in the PICU with peripheral access, the dwell times and the modifying parameters have not been reported. The mean dwell time was found to be 50.1 h (measured from insertion to removal) that aligns well with the mean dwell time previously reported for hospitalized children (55.6–67 h) (5, 14). The median duration of functional dwell time from insertion to documented failure computed using non-parametric “survival” analysis was 82 h (IQR: 67.7–97.2). The potential for inflation may arise due to delays in discovery or documentation issues.

Increased dwell time was associated with female gender 59.5 h (*P *< 0.001), first attempt insertion 53.5 h (*P *< 0.001), use of 24 Ga bore 56.3 h (*P *= 0.04), left-sided insertions 54.9 h (*P *= 0.07), less patient agitation 54.8 h (*P *= 0.02), and less edema 61.6 h (*P *< 0.001). None of the observed increases exceeded 1.5 times the average dwell time (1.5 × 50.1 h = 75.2 h). Therefore, even under favorable conditions that allow for longer durations, the dwell time remains shorter according to the practitioner’s preferences. A decreased dwell time was associated with the use of vancomycin infusion at 24.2 h (*P* < 0.001) and blood transfusions at 29.3 h (*P* < 0.001).

It was observed that if a patient had repeated insertions, the subsequent insertion of PIVCs in the admission course lasted longer. The number of repeated insertions is correlated strongly (Pearson *ρ *=* *0.89) with EDC and MC insertion. Following the insertion of another type of vascular access, the PIVC may be used less frequently. For example, a patient with a midline may require a PIVC for occasional additional medications. The PIVC will last longer than when utilizing it for all the infusions. A third insertion increases the odds of vascular access team consultation and EDC or MC placement to 5.2.

A recent literature showed that PIVCs can be used for inotropes, 3% saline, and TPN solutions (15–18). The data presented in this study could not confirm or reject the hypothesis, as it did not include an evaluation of such a hypothesis. However, it was observed that occasional PIVCs (6.6% of the total) were utilized for inotropes, hypertonic saline, and TPN solutions for a duration of less than 24 h. It is important to note that the dopamine was diluted for peripheral use, the hypertonic saline was administered at a concentration of only 2%, and the TPN solution contained dextrose up to a 12.5% concentration as per institutional policy.

It is still unclear whether the PIVCs might be equally responsible for thrombosis and catheter infections; some reports already implicate midlines. In a published study, the rate of EDC-associated infection varied from 0 to 1.07/1,000 midline days (19–22). The studies supported that “silicone-base” catheters such as PICC have a lower incidence of infection (21). CVCs were associated with a higher risk of central line-associated bloodstream infection (CLABSI) (RR = 2.20; 95% CI: 1.05–4.61; *P *= 0.04) compared with PICCs. The incidence of thrombosis was reported to be higher in midline catheters than in PICCs (7.04% MCs and 4.72% PICCs; OR: 1.53; *P *= 0.01), although the incidence is low and requires further studies to be validated (22). Currently, there is a lack of available data regarding the incidence of thrombosis or infection associated with EDCs.

Uncomplicated vascular access is an illusion. A fine-tuning of the treatment options is required in order to achieve a balance between complications. According to the Centers for Disease Control and Prevention (CDC), it is recommended to utilize an MC or PICC catheter when administering therapy that extends beyond 60 days (https://www.cdc.gov/infectioncontrol/guidelines/bsi/recommendations.html). The miniMAGIC recommendations offer guidelines based on the expected duration of the therapy (7 days). PIVC-based treatment for 6–7 days can potentially result in several painful insertion attempts due to their relatively short dwelling time, and the infectious complications (if any, since they have not been reported for EDCs) should be carefully evaluated in relation to the recurring adverse effects of PIVCs. Multiple attempts increase the chances of infections, phlebitis, and thrombosis risks. Repeated attempts can potentially cause infiltration/extravasation from the previously injured vessel (22–24). Since the odds of EDC/MC increase after the third insertion, it is advisable to prioritize their early consideration and subsequent insertion without exposing the patient to the discomfort of repeated insertions (23).

This study did not specifically evaluate the success rate of insertion using different methods—the vascular access team using ultrasounds to implant all the EDC, MD, and PICC in the authors’ institution. Vascular access teams have been associated with a reduction in CLABSI and an improvement in patient satisfaction (25, 26). A 2018 Cochrane review suggested that there is a need for further research on the utilization of vascular access teams (27, 28).

A comprehensive analysis of 3,700 US-based hospitals provided a detailed causal-comparative design that addressed the difference in the reported CLABSI rates based on hospital type (teaching and non-teaching) for hospitals with a vascular access team and whether there was a difference in the reported CLABSI rates based on the presence or absence of a vascular access team (29). The results demonstrated there were significant differences in the reported CLABSI rates by hospital type (*P *< 0.001), and the presence or absence of a VA team was also of consequence, indicating that there were significant differences in reported CLABSI rates (*P *= 0.004) based on the presence or absence of a VA team (29).

The results suggested that a dedicated VA team is associated with a lower incidence of infection, regardless of hospital type. This finding is consistent with the results of previous studies showing that VA teams can improve patient outcomes by reducing the risk of infection, complications, and costs associated with vascular access devices. The findings of this study will guide healthcare leaders in their efforts to implement evidence-based guidelines and infection risk reduction strategies throughout healthcare organizations. Establishing VA teams inside healthcare organizations has the potential to improve patient safety and increase the quality of care provided to those in need of vascular access.

Accurately monitoring peripheral vascular access, dwell time, and complication is a vital source of information, and it has to be communicated in a non-penalizing way to encourage accurate reporting. The authors do not support the practice of utilizing peripheral access in critical care patients as a means of reducing the incidence of central access complications. Instead, they advocate for an evidence-based evaluation of vascular access that takes into account the specific requirements for each patient. Educational competency in peripheral vascular accesses is also essential. and performing an ultrasound-guided placement of central catheter is a crucial aspect of this competency (30).

The recommended duration of treatment was outlined in the miniMAGIC guidelines. This study suggests using EDC in cases when there is edema, agitation, or a need for vancomycin infusion, transfusion, or prolonged treatment. The scope of this study is limited to a retrospective, single-center analysis. The ability of the PIVC placement varies with the experience, care ratio, and specific infusion policies. The limitations of dwell time reporting have already been highlighted. The actual dwell times observed in the sample depend on the specific characteristics of the PICU, but the underlying relations are expected to remain similar. The experience of the team and various protocols for early insertion will increase the dwell time. The miniMAGIC system covers specifically PICU patients only, but it is particularly beneficial for pediatric patients with congenital heart disease who often require femoral access for catheterization; in these cases, the EDC option is even more advantageous.

## Conclusions

EDC/MC is a superior alternative to PIVCs for peripheral access in a considerable number of PICU patients. The need for vascular access consultation and more stable insertion increased following the third PIVC. The absence of a skilled clinician, resulting in the occurrence of multiple punctures, has the potential to cause injury to patients. A third insertion might be a simple bedside criterion to alert the team to request a consultation for an overdue EDC or MC. However, early evaluation and utilization of vascular access teams are considered the optimal clinical approach.

The recommendation of miniMAGIC protocols is sufficient for central vascular access. For peripheral access, the duration of the treatment should also be considered. Comfort, fewer painful punctures, and preventing complication from vancomycin and transfusions are essential. Extended dwell catheters and vascular team consultation offer a great alternative to PIVCs. The study recommends that healthcare providers should take into account the patient's edema, the reported or estimated insertion difficulty, the level of agitation, and the need for transfusion or vancomycin infusion for an early call for EDC in PICU patients. This approach may prove advantageous even for patients with a treatment duration of less than 6 days.

## Data Availability

The raw data supporting the conclusions of this article will be made available by the authors, without undue reservation.
